# Consequences of hypomagnesemia in type 2 diabetes mellitus patients

**DOI:** 10.12861/jrip.2014.28

**Published:** 2014-12-01

**Authors:** Hamid Nasri

**Affiliations:** Department of Nephrology, Division of Nephropathology, Isfahan University of Medical Sciences, Isfahan, Iran

**Keywords:** Magnesium, Diabetes mellitus, Dyslipidemia

**Implication for health policy/practice/research/medical education:**

Clinical care should therefore focus on increasing dietary magnesium intake or magnesium supplementation to improve metabolic control and prevent dyslipidemia in 2 diabetes mellitus patients.

Magnesium (Mg) is the fourth most plentiful mineral and encountered as the second most abundant intracellular divalent cation and has been accepted as a cofactor for >300 metabolic reactions in in humans (1). Around 50% of this element is in the bone tissue, 50% is in the tissues and organs, and 1% is in the blood (1,2). Mg is a significant cofactor in a number of key enzymatic reactions and seems to play a vital role in glucose metabolism and insulin homeostasis (2-4). Recently, various evidences suggesting a relationship of Mg deficiency and type 2 diabetes mellitus (T2DM). T2DM is a main global public health problem in the world and is rising in aging populations (4,5). In the study conducted by Dasgupta *et al*. on 150 T2DM patients, low Mg level was documented in 11.33% of patients. They interpreted that hypomagnesemia in diabetes mellitus was correlated with poorer diabetic control, nephropathy, retinopathy and foot ulcers (6). Likewise, Baig *et al*. conducted an investigation on 60 T2DM patients between 40 and 70 years of age. They detected that the mean serum Mg value was significantly low in T2DM patients without and with complications when compared with control group. Additionally, they showed that serum Mg value in cases with diabetic complications was significantly lower than those without complications (7). We also previously conducted a study to test the association of serum lipids with serum Mg in T2DM patients in 2008. In this study, on 122 T2DM patients, we found a significant inverse associations of serum Mg value with serum cholesterol and low-density lipoprotein cholesterol (LDL-C) (8). Recently, Kocot *et al*. carried-out a study on 54 T2DM. They found low serum value of Mg in diabetic individuals in comparison to healthy participants. They also detected a weak negative correlation between plasma magnesium level and total cholesterol and also between plasma Mg and serum triglyceride in diabetic patients (9). The result of this study was in accord with our previous results. More recently Mishra *et al*. examined 45 known T2DM patients. They observed significant negative association of serum Mg level with triglyceride and very low-density lipoprotein cholesterol level and positive association of Mg with serum high-density lipoprotein cholesterol too (10). The association of hypomagnesemia and insulin resistance in diabetes patients has been documented previously. Accordingly in the study of 219 T2DM patients (11-13). Rasheed *et al*. observed serum Mg had significantly positive association with serum high-density lipoprotein cholesterol while total cholesterol and very low-density lipoprotein cholesterol was negatively correlated, though non-significantly, with serum Mg (14). Moreover, a study on 550 diabetic patients, revealed serum Mg, significant negative correlation with glomerular filtration rate (15).

## Conclusion

Clinical care should therefore focus on increasing dietary Mg intake or Mg supplementation to improve metabolic control and prevent dyslipidemia in T2DM patients.

## Author’s contribution

HN is the single author of the paper.

## Conflict of interests

The author declare that there were no conflicts of interest.

## Ethical considerations

Ethical issues (including plagiarism, data fabrication, double publication) have been completely observed by the author.

## Funding/Support

None.

## References

[1] Elin RJ (1994). Magnesium: the fifth but forgotten electrolyte. Am J Clin Pathol.

[2] Takaya J, Higashino H, Kobayashi Y (2004). Intracellular magnesium and insulin resistance. Magnes Res.

[3] Wild S, Roglic G, Green A, Sicree R, King H (2004). Global prevalence of diabetes: estimates for the year 2000 and projections for 2030. Diabetes Care.

[4] Arnaud MJ (2008). Update on the assessment of magnesium status. Br J Nutr.

[5] Niranjan G, Anitha D, Srinivasan AR, Velu VK, Venkatesh C, Babu MS (2014). Association of inflammatory sialoproteins, lipid peroxides and serum magnesium levels with cardiometabolic risk factors in obese children of South Indian population. Int J Biomed Sci.

[6] Dasgupta A, Sarma D, Saikia UK (2012). Hypomagnesemia in type 2 diabetes mellitus. Indian J Endocrinol Metab.

[7] Baig MS, Shamshuddin M, Mahadevappa KL, Attar AH, Shaikh AK (2012). Serum magnesium as a marker of diabetic complications. J Evol Med Dent Sci.

[8] Nasri H, Baradaran HR (2008). Lipids in association with serum magnesium in diabetes mellitus patients. Bratisl Lek Listy.

[9] Kocot DL, Sztanke M, Wiśniewska MN, Dąbrowski A, Andrzejewski A, Wallner G (2012). Plasma magnesium and calcium concentrations and selected biochemical parameters in patients with type 2 diabetes mellitus. Curr Issues Pharm Med Sci.

[10] Mishra S, Padmanaban P, Deepti GP, Sarkar G, Sumathi G, Toora BD (2012). Serum magnesium and dyslipidemia in type-2 diabetes mellitus. Biomed Res.

[11] Chiarello DI, Marín R, Proverbio F, Benzo Z, Piñero S, Botana D (2014). Effect of hypoxia on the calcium and magnesium content, lipid peroxidation level, and Ca²^+^-ATPase activity of syncytiotrophoblast plasma membranes from placental explants. Biomed Res Int.

[12] Srinivasan AR, Niranjan G, Kuzhandai Velu V, Parmar P, Anish A (2012). Status of serum magnesium in type 2 diabetes mellitus with particular reference to serum triacylglycerol levels. Diabetes Metab Syndr.

[13] Volpe SL (2008). Magnesium, the metabolic syndrome, insulin resistance, and type 2 diabetes mellitus. Crit Rev Food Sci Nutr.

[14] Rasheed H, Elahi S, Ajaz H (2012). Serum magnesium and atherogenic lipid fractions in type II diabetic patients of Lahore, Pakistan. Biol Trace Elem Res.

[15] Pham PC, Pham PM, Pham PT (2012). Patients with diabetes mellitus type 2 and hypomagnesemia may have enhanced glomerular filtration via hypocalcemia. Clin Nephrol.

